# Dehydration of Organic Solvents from Ternary Mixtures Containing Toluene/Methanol/Water by Pervaporation

**DOI:** 10.3390/membranes14060139

**Published:** 2024-06-12

**Authors:** Ying Qiao, Shichang Xu, Yixuan Wu, Long Zhang, Lixin Xie

**Affiliations:** Tianjin Key Laboratory of Membrane Science and Desalination Technology, State Key Laboratory of Chemical Engineering, School of Chemical Engineering and Technology, Tianjin University, Tianjin 300350, China; qiaoying@tju.edu.cn (Y.Q.); wuyx@tju.edu.cn (Y.W.); zhanglong123@tju.edu.cn (L.Z.)

**Keywords:** dehydration of organic solvents, ternary mixtures, permeate flux, separation factor, pervaporation

## Abstract

The separation of a toluene/methanol/water ternary mixture is a difficult task due to the toluene/water and toluene/methanol azeotropes. In this article, low-energy pervaporation is proposed for the separation of the ternary azeotrope toluene–methanol–water. This work investigates the effects of feed temperature, feed flow rate, and vacuum on pervaporation and compares the energy consumption of pervaporation with that of distillation. The results showed that at the optimized flow rate of 50 L/h and a permeate side vacuum of 60 kPa at 50 °C, the water and methanol content in the permeate was about 63.2 wt.% and 36.8 wt.%, respectively, the water/ methanol separation factor was 24.04, the permeate flux was 510.7 g/m^2^·h, the water content in the feed out was reduced from 2.5 wt.% to less than 0.66 wt.%, and the dehydration of toluene methanol could be realized. Without taking into account the energy consumption of pumps and other power equipment, pervaporation requires an energy consumption of 43.53 kW·h to treat 1 ton of raw material, while the energy consumption of distillation to treat 1 ton of raw material is about 261.5 kW·h. Compared to the existing distillation process, the pervaporation process consumes much less energy (about one-sixth of the energy consumption of distillation). There is almost no effect on the surface morphology and chemical composition of the membrane before and after use. The method provides an effective reference for the dehydration of organic solvents from ternary mixtures containing toluene/methanol/water.

## 1. Introduction

With the rapid development of the chemical industry, organic matter refining has become more and more important in petrochemicals, fine chemicals, organic chemicals, pharmaceutical chemicals, and other fields; one problem is the dewatering of organic matter [1]. A typical organic mixture is toluene, methanol, and water, which are often formed in chemical and pharmaceutical processes. Toluene and methanol can be recycled in production processes to save costs. However, the separation of methanol/toluene/water mixtures is a major challenge due to the toluene azeotropes formed with both methanol and water. Extractive distillation [2,3] and azeotropic distillation [4] processes are common methods for separating methanol/toluene/water mixtures. However, these processes usually require a lot of energy. Therefore, energy-efficient methods for separating methanol/toluene/water mixtures are still needed.

In 2018, Wang et al. [5] proposed a double-column extractive distillation process with a decanter for the separation of a ternary azeotropic mixture. The system employed NMethyl-2-pyrrolidone (NMP) as the solvent. Methanol was recovered from the upper section of the initial extractive distillation column, while the bottom stream containing toluene, water, and NMP was directed to another column. Toluene and water were then distilled from the top of the second column and subsequently separated into a toluene phase and a water phase with a decanter. Although extraction distillation can achieve the separation of an azeotropic mixture, the remixing effect during extraction distillation leads to a lot of energy waste. Cui et al. [4] proposed a liquid side-stream extractive distillation process for the separation of benzene/isopropanol/water. The results showed that although double liquid side-stream extraction distillation could effectively reduce the total annual cost, the energy consumption was too high. Therefore, the pervaporation process has attracted the attention of researchers in recent years.

Nowadays, the main methods for dehydrating organic matter include distillation [6], gas extraction [7], adsorption [8,9], and membrane separation [10,11]. Among them, membrane separation is considered a new dehydration method that has been used since the end of the 20th century. Membrane separation offers the advantages of high separation performance, low energy consumption, and environmental protection.

Pervaporation is a membrane-based separation method that uses a selectively permeable membrane as a separation medium to separate, sort, and enrich the components of a mixture. It is suitable for the separation of components and azeotropes [12,13] in organic mixtures [14], mixtures with similar boiling points [15], and heat-sensitive compounds [16]. It offers the advantages of energy efficiency, small footprint, scalability, and easy integration [17,18]. Currently, pervaporation is used to separate binary systems, including toluene/methanol [19] and toluene/water [20,21]. However, to the best of our knowledge, there are only a few studies on the pervaporation of ternary methanol/toluene/water mixtures.

Yi et al. [8] used a hydrophilic chitosan-modified polybenzimidazole (PBI) membrane for the percolation dehydration of an aqueous isopropyl alcohol solution. When the water content in the raw material liquid was 30% and the temperature was 70 °C, the maximum membrane flux was 250 g/(m^2^·h) and the maximum separation factor was 115. Zhang et al. [22] prepared a ceramic cavities fiber membrane with poly4-styrene sulfonic acid and chitosan as raw materials through a multilayer assembly process and performed pervaporation separation of the aqueous ethanol solution. At a water content of 10%, the flux of the membrane was 495 g/(m^2^·h) and the separation factor was 904 at 70 °C. Chen et al. [23] prepared polyelectrolyte complex/silica (PEC/SiO_2_) hybrid hollow fiber membranes for the dehydration of fusel oils. The results showed that the membranes had a flux of 1332 g/(m^2^·h) and a water content in the permeate of 99.0 wt.% for the dehydration of fusel oils at 60 °C. Tong et al. [24] exploited the synergistic effect of polyelectrolyte (PEL) and sulfonated graphene oxide (SGO) to fabricate novel composite membranes and achieve the ultrafast transport of water molecules in isopropanol pervaporation dehydration. The results showed that at 70 °C, the flux could reach up to 3.67 kg/(m^2^·h) with a water content of 99.90 wt.% in the permeate. Mohammadi et al. [25] prepared alkali-activated ceramic membranes (AACMs) for ethanol dehydration. The results showed that the optimal membrane had a pervaporation separation index (PSI), permeate flux, and separation factor of 954.52, 3.61 kg/(m^2^·h), and 264.41, respectively, in ethanol dehydration by pervaporation.

In this work, we used a low-energy-consumption pervaporation strategy to treat the dehydration of methanol/toluene/water mixtures. We optimized the operating parameters of feed temperature, feed flow rate, and vacuum during pervaporation. The surface morphology and chemical composition of the PVA membranes were tested before and after 80 days (about 1500 h) of use to investigate their stability in the experimental system. A comparison of the energy consumption of pervaporation and distillation was then carried out. The method provides an effective reference for the dehydration of organic solvents from ternary mixtures containing toluene/methanol/water.

## 2. Experiment

### 2.1. Materials

Toluene and anhydrous methanol (AR, Tianjin Kemiou Co., Ltd., Tianjin, China) were used in the experiments. All the water used in this study was DI water (conductivity < 10 μS·cm^−1^). The PVA membrane was purchased from Shandong Lanjing Membrane Technology Engineering Co., Ltd., Tai’an, China. The thickness of the PVA membrane was 170 μm, the pore size was 10 nm, the molecular weight was 100 kDa, the pH was 6.5–8.5, and the temperature was not more than 90 °C. The experimental organic solvent consisted of 62.5 wt.% toluene, 35.0 wt.% methanol, and 2.5 wt.% water.

### 2.2. Pervaporation Experiments

**Figure 1:**
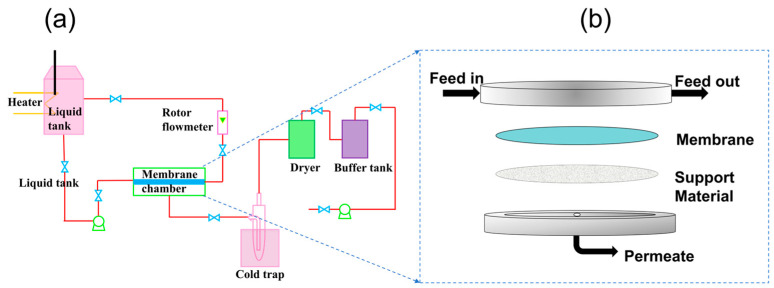
Schematic diagram of (**a**) lab-scale pervaporation configuration and (**b**) membrane cell.

The pervaporation experiment was carried out with the same equipment used in a previous report [26], as shown in Figure 1a. The schematic diagram of the membrane cell is shown in Figure 1b. The laboratory device was a P-28 membrane unit from CM-Celfa Membrantechnik AG (Zurich, Switzerland) with an effective area of 25.6 cm^2^ (A). The size of the inlet tank was 2 L. The concentrations of toluene, methanol, and water were determined by a gas chromatography system (Agilent 6820, Santa Clara, CA, USA) equipped with a 3.0 m long column filled with PEG 20M and TCD detector (Switzerland), with a column temperature of 373 K.

The membrane was cut according to the size of the porous support screen plate and the membrane module was assembled as shown in Figure 1b. Toluene methanol and water were added into the raw material tank, the circulation valve and gear pump were opened to circulate the material liquid at a flow rate of 50 L/h [27], and the temperature control system was opened to keep the material liquid temperature at 50 °C, The empty cold hydrazine was weighed and when the temperature of the raw material liquid and circulation was stabilized, a cold trap was connected to the downstream side of the membrane chamber, the vacuum pump was opened, and the cold hydrazine was immersed in liquid nitrogen for 3 min. The valve on the downstream side was opened and a timer was started. The cold trap containing the permeate was thawed, weighed when the permeate had melted, and analyzed by GC. After the test was completed, the temperature control system and vacuum device were closed and the raw material liquid was allowed to drop to room temperature. Then, the circulation device was closed, the membrane module was unloaded, and the experiment was ended.

### 2.3. Characterizations of PVA Membrane

#### 2.3.1. Contact Angle

In this study, a Powereach JC2000C static contact angle tester (Shanghai Zhongchen Digital Technology Equipment Co., LTD., Shanghai, China) was used to measure the contact angle of the composite film surface. The prepared membrane sample was placed on the platform, the detection liquid (deionized water) was dropped onto the surface of the membrane using a microsyringe to form a droplet with a diameter of about 5 mm, and the corresponding contact angle was determined. For precise determination, 3 parallel samples were taken from each sample and 6 different test points were selected on each parallel sample. The mean of the test results was used.

#### 2.3.2. Measurement of Membrane Swelling

The cut PVA membrane was placed in a vacuum drying oven at 40 °C for 2 h and then taken out. The mass was quickly weighed with an electronic balance and recorded as *W*_d_ and then immersed in a certain concentration of toluene methanol aqueous solution at a constant temperature to reach the equilibrium of swelling (weighed several times as a constant weight; it was concluded that the equilibrium of swelling took 24 h). It was then taken out and the liquid on the surface was quickly dried with filter paper and weighed as *W*_s_. Then, the equilibrium solubility of the membrane in aqueous toluene methanol, i.e., *D*_s_ (Degree of Swelling) was calculated by the following equation:

(1) $$D_{s}=\frac{W_{s}-W_{d}}{W_{d}}\times 100\%$$

### 2.4. Data Analysis

The *W*_i_% calculation formula was as follows:

(2) $$W_{i}\%=\frac{fiAi}{\sum_{i=1}^{n}fiAi}\times 100\%$$

where *W*_i_ is the mass fraction of component i, *f*_i_ is the correction factor for component i, and *A*_i_ is the peak area of component i.

The permeate flux J was calculated by the following equation:

(3) $$J=\frac{Q}{A\times t}$$

where *Q* represents the weight of the permeant, *A* is the effective membrane area, and *t* is the operation time.

The separation factor was defined as below:

(4) $$\alpha =\frac{y_{w}/y_{m}}{x_{w}/x_{m}}$$

where *y*_w_ is the weight fraction of water in the permeate, *y*_m_ is the weight fraction of methanol in the permeate, *x*_w_ is the weight fraction of water in the feed, and *x*_m_ is the weight fraction of methanol in the feed.

Permeate flux *J* was defined as below:

(5) $$LnJ=LnJ_{0}-\frac{E_{J}}{RT}$$

where *J* is the permeate flux, *J*_0_ is the pre-exponential factor, *E*_J_ is the apparent activation energy, *T* is the feed temperature, and *R* is the gas constant.

Pervaporation for the energy consumption calculation [28] was as follows:

(6) $$Q_{pevap}=Q∆H_{ev}$$

where *Q*_pevap_ is pervaporation energy, *Q* represents the weight of the permeant, and Δ*H*_ev_ is the latent heat of evaporation.

## 3. Results and Discussion

### 3.1. Influence of Operating Conditions on Pervaporation

#### 3.1.1. Influence of Feed Temperature

Feed temperature was one of the important factors affecting the pervaporation process. It could affect the permeate flux and separation factor in the pervaporation process by affecting the solubility and diffusion coefficient of each component in the liquid in the membrane.

**Figure 2:**
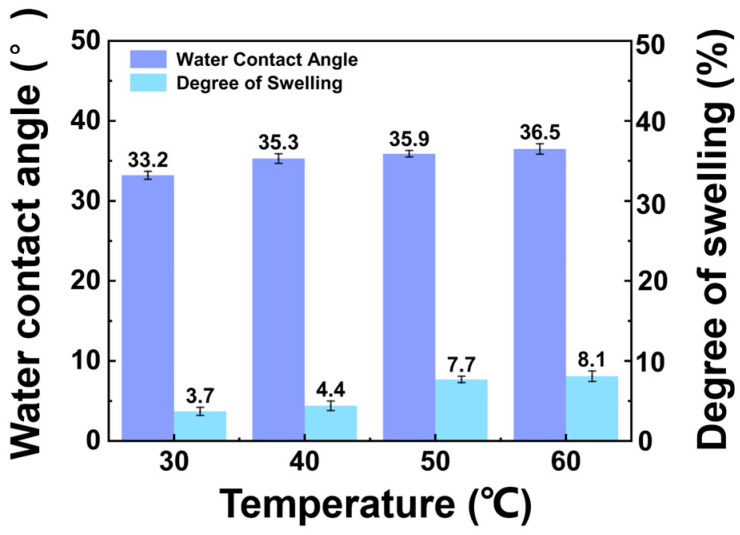
Effect of temperature on the water contact angle and degree of swelling. (The experimental organic solvent was 62.5 wt.% toluene, 35.0 wt.% methanol, and 2.5 wt.% water.)

The water contact angle of the PVA membrane is shown in Figure 2. The water contact angle of the membrane increased with temperature [29]. The PVA exhibited high hydrophilicity, with all water contact angles being less than 37°. The degree of swelling of the PVA membrane in the feed solution is also shown in Figure 2. The swelling of the membrane increased with temperature [30]. This may have been because the increase in temperature provided energy to the polymer chain segments within the membrane and the molecules in the immersion solution, accelerating their movement. All degrees of swelling were below 10%, meeting the application criteria of the pervaporation process.

**Figure 3:**
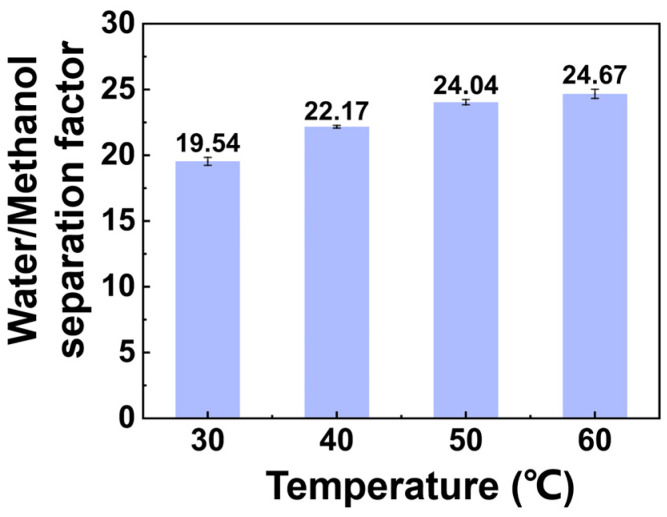
Effect of temperature on separation factor. (The experimental organic solvent was 62.5 wt.% toluene, 35.0 wt.% methanol, and 2.5 wt.% water and the pervaporation test was conducted with a constant vacuum degree of 60 kPa and flow rate of 50 L/h.)

The separation factor at different temperatures is shown in Figure 3. The water/methanol separation factor increased with increasing temperature. Increased temperature led to weak adhesion between molecules, which led to an increase in the molecular diffusion rate, thereby increasing the speed of molecules passing through the membrane.

**Figure 4:**
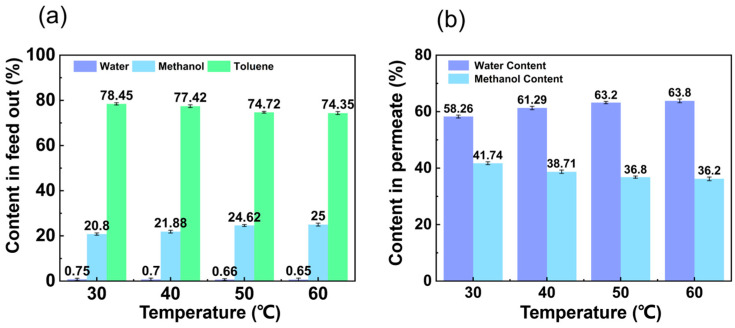
Effect of temperature on (**a**) content in feed out and (**b**) content in permeate. (The experimental organic solvent was 62.5 wt.% toluene, 35.0 wt.% methanol, and 2.5 wt.% water and the pervaporation test was conducted with a constant vacuum degree of 60 kPa and flow rate of 50 L/h.)

The content in the feed out at different temperatures is shown in Figure 4a. The water content in the feed out decreased with temperature from 0.75 wt.% to 0.65 wt.%. The toluene content in the feed out also decreased with temperature from 78.45 wt.% to 74.35 wt.%. In contrast, the methanol content in the feed out increased with temperature from 20.80 wt.% to 25.00 wt.%. The mass transport of pervaporation in the PVA can be explained by the dissolution–diffusion mechanism. Methanol and water have better hydrophilicity than toluene and can be preferentially dissolved in hydrophilic PVA. From Figure 4b, it can be seen that the water content in the permeation solution increased with increasing temperature. In contrast, the methanol content in the permeable solution decreased from 41.74 wt.% to 36.20 wt.%, which fully indicates that the membrane preferentially adsorbed and dissolved water and had preferential permeability for water.

**Figure 5:**
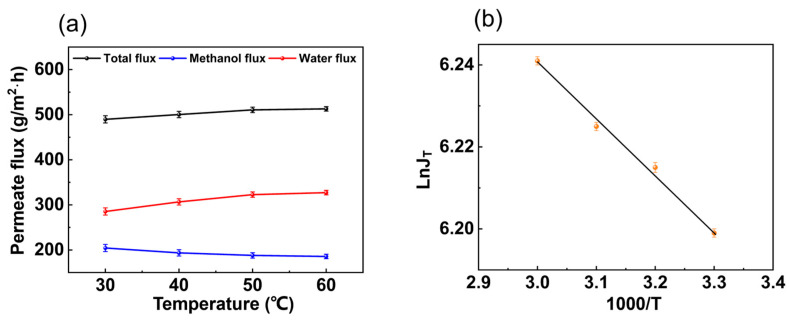
Effect of temperature on (**a**) permeate flux and (**b**) LnJ_T_ and 1000/T. (The experimental organic solvent was 62.5 wt.% toluene, 35.0 wt.% methanol, and 2.5 wt.% water and the pervaporation test was conducted with a constant vacuum degree of 60 kPa and flow rate of 50 L/h.)

In Figure 5a, from 30 °C to 60 °C, the total flux and water flux increased with temperature, while the methanol flux showed the opposite trend. As the feed temperature increased, the partial pressure difference between the upstream and downstream sides of the membrane increased accordingly, which improved mass transfer. The change in the permeate flux of the membranes can be described by the Arrhenius equation, and the apparent activation energy can be described according to Equation (5). In Figure 5b, LnJ_T_ and 1000/T can be fitted as a straight line. The average value of the osmotic activation energy of the membrane was *E*_J_ = 1.745 kJ/mol.

#### 3.1.2. Influence of Feed Flow Rate

**Figure 6:**
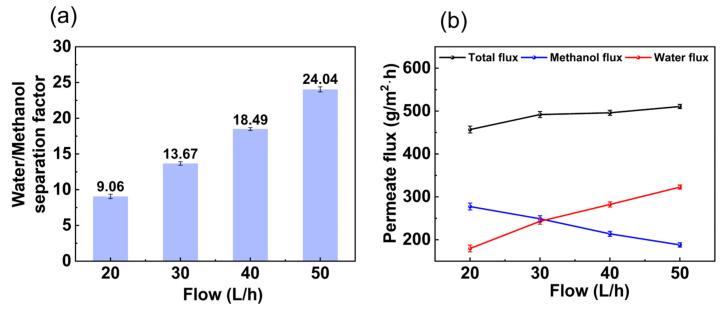
Effect of flow rate on (**a**) separation factor and (**b**) permeate flux. (The experimental organic solvent was 62.5 wt.% toluene, 35.0 wt.% methanol, and 2.5 wt.% water and the pervaporation test was conducted with a constant vacuum degree of 60 kPa and at (50 ± 1) °C.)

The influence of feed flow rate on membrane separation performance affected the flow state on the membrane surface. As shown in Figure 6a, at a flow rate between 20 L/h and 50 L/h, the water/methanol separation factor increased with the flow rate. The increased feed flow rate increased the turbulence of the liquid phase to some extent, and the boundary separation membrane on the membrane surface was compressed by the high flow rate, which weakened the concentration polarization phenomenon and strengthened the mass transfer driving force of the concentration gradient to some extent. As shown in Figure 6b, the total flux and water flux increased with the flow rate from 20 L/h to 50 L/h, while the methanol flux showed the opposite trend. Increasing the flow rate effectively reduced the concentration polarization and thus improved the overall permeate flux.

**Figure 7:**
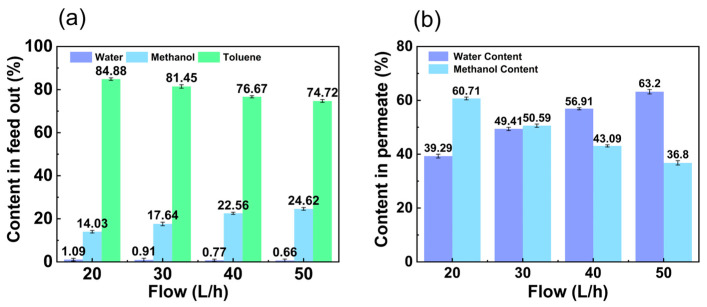
Effect of flow rate on (**a**) content in feed out and (**b**) content in permeate. (The experimental organic solvent was 62.5 wt.% toluene, 35.0 wt.% methanol, and 2.5 wt.% water and the pervaporation test was conducted with a constant vacuum degree of 60 kPa and at (50 ± 1) °C.)

The contents of the feed out at different flow rates are shown in Figure 7a. The water content in the feed out decreased with flow rate from 1.09 wt.% to 0.66 wt.%. The toluene in the feed out also decreased with flow rate from 84.88 wt.% to 74.72 wt.%. In contrast, the methanol content in the feed out increased with flow rate from 14.03 wt.% to 24.62 wt.%. From Figure 7b, it can be seen that the water content in the permeate increased with increasing feed flow rates from 39.29 wt.% to 63.20 wt.%, while the methanol content showed the opposite trend, decreasing from 60.71 wt.% to 36.80 wt.%. As the flow rate increased, the state of the fluid gradually approached turbulence, creating better contact between the fluid and the membrane. At the same time, the interaction between the liquid and the membrane was enhanced, which improved the transfer of permeable components through the membrane.

#### 3.1.3. Influence of Vacuum

**Figure 8:**
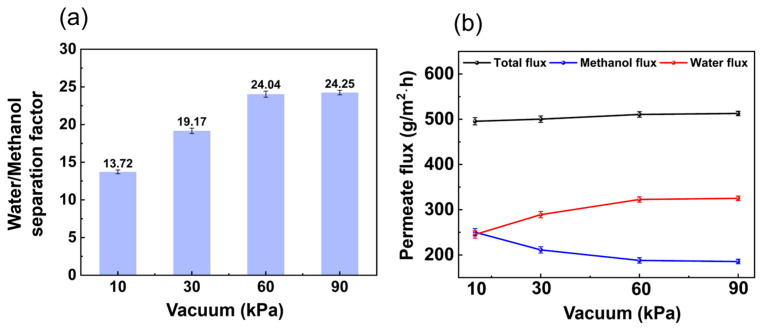
Effect of vacuum on (**a**) separation factor and (**b**) permeate flux. (The experimental organic solvent was 62.5 wt.% toluene, 35.0 wt.% methanol, and 2.5 wt.% water and the pervaporation test was conducted with a flow rate of 50 L/h and at (50 ± 1) °C.)

In Figure 8a, with a vacuum between 10 kPa and 90 kPa, the separation factor of water/methanol increased with vacuum degree. In fact, increasing the driving force resulted in a faster desorption rate of the adsorbed molecules. The vapor pressure of the components of the feed mixture determined the membrane selectivity. The water/methanol separation factor therefore increased with increasing vacuum degree. In Figure 8b, the total flux and water flux increased with vacuum degree from 10 kPa to 90 kPa, while the methanol flux showed the opposite trend. The reason for this is that as the vacuum degree increased, the pressure difference between the front and back of the membrane increased, the driving force of pervaporation increased, and the overall permeate flux increased.

**Figure 9:**
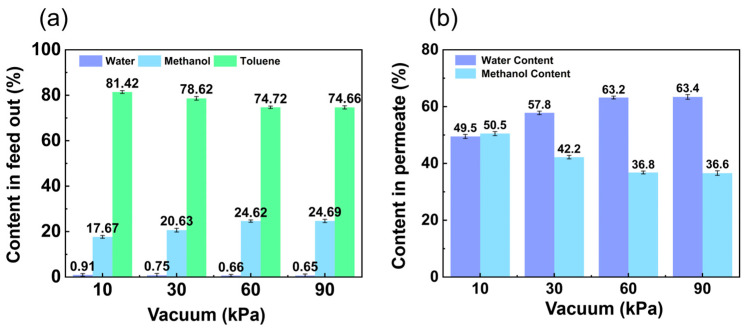
Effect of vacuum on (**a**) content in feed out and (**b**) content in permeate. (The experimental organic solvent was 62.5 wt.% toluene, 35.0 wt.% methanol, and 2.5 wt.% water and the pervaporation test was conducted with a flow rate of 50 L/h and at (50 ± 1) °C.)

The contents of the feed out with different vacuums are shown in Figure 9a. The methanol content in the feed out increased with vacuum degree from 17.67 wt.% to 24.69 wt.%. In contrast, the toluene in the feed out decreased with vacuum force from 81.42 wt.% to 74.66 wt.% and the water content in the feed out also decreased with vacuum force from 0.91 wt.% to 0.65 wt.%. From Figure 9b, it can be seen that the water content in the permeate increased from 49.50 wt.% to 63.40 wt.% with the increase in vacuum degree, while the methanol content showed the opposite trend, decreasing from 50.50 wt.% to 36.60 wt.%.

### 3.2. Operational Stability of Pervaporation

**Figure 10:**
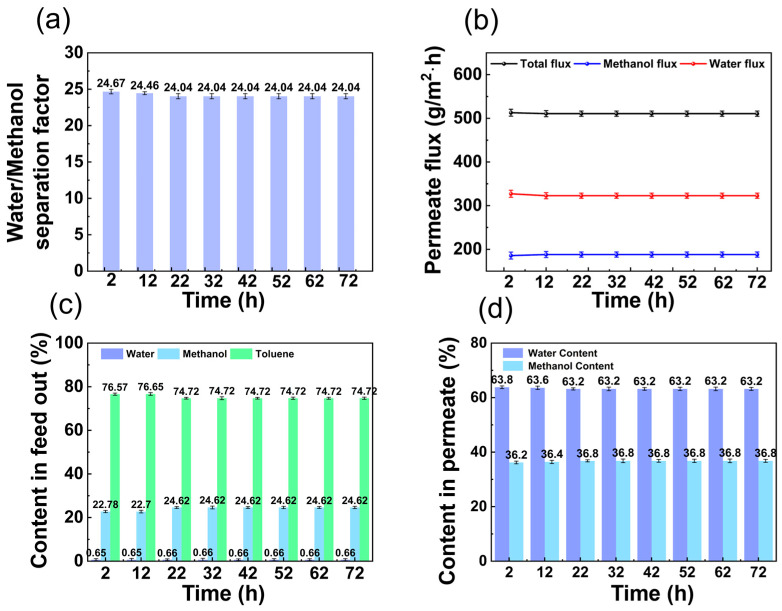
Continuous-mode experiments by pervaporation: (**a**) separation factor; (**b**) permeate flux; (**c**) content in feed out; (**d**) content in permeate. (The experimental organic solvent was 62.5 wt.% toluene, 35.0 wt.% methanol, and 2.5 wt.% water and the pervaporation test was conducted with a constant vacuum degree of 60 kPa, at (50 ± 1) °C, and with a flow rate of 50 L/h.)

The PVA membrane was subjected to operational stability experiments for up to 72 h under the experimental conditions of 50 °C, flow rate of 50 L/h, and permeate side vacuum degree of 60 kPa. As shown in Figure 10a, the water/methanol separation factor was 24.04. As shown in Figure 10b, the total flux was about 510.7 g/m^2^·h, the water flux was about 322.7 g/m^2^·h, and the methanol flux was about 188.0 g/m^2^·h. As shown in Figure 10c, the water, methanol, and toluene contents in the feed out were about 0.66 wt.%, 24.62 wt.%, and 74.72 wt.%, respectively. As shown in Figure 10d, the water content in the permeate was maintained at 63.2 wt.%, showing good operational stability in the test.

### 3.3. PVA Membrane Properties Analysis

#### 3.3.1. SEM Analysis

**Figure 11:**
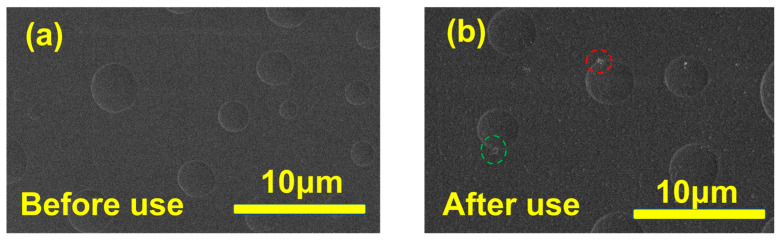
Surface SEM images of PVA (**a**,**b**). The morphology of the membrane before and after 80 days of use in (**a**,**b**), respectively.

The surface morphology of the PVA membranes was tested before and after 80 days (about 1500 h) of use to investigate their stability in the experimental system. Scanning electron microscopy was used to observe the changes in the surface morphology of the membrane before and after use. The results are shown in Figure 11. After 1500 h of operation, the surface of the PVA membrane was still relatively compact and flat, and there was no obvious change in the membrane structure.

#### 3.3.2. FT-IR Analysis

**Figure 12:**
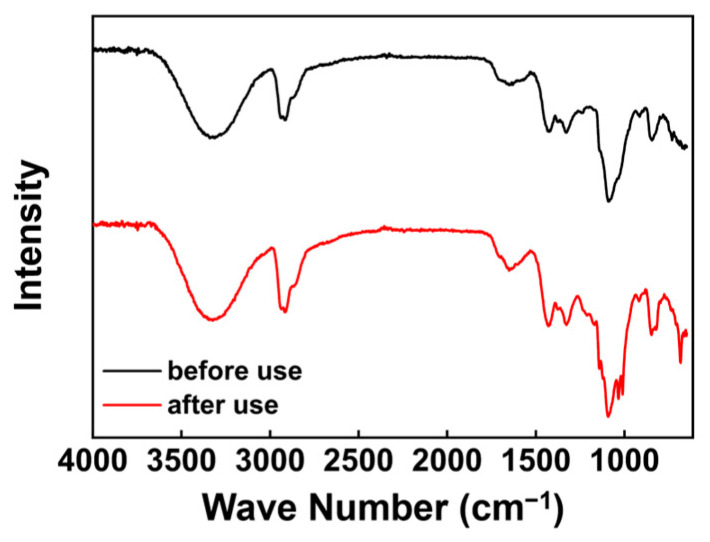
FT-IR images of PVA membrane.

The chemical composition of the PVA membranes was tested before and after 80 days (about 1500 h) of use to examine their stability in the experimental system. The results of the infrared spectrum test are shown in Figure 12. It can be seen from the figure that the position and content of the peaks on the surface of the PVA membrane did not change significantly before and after use, indicating that the use of 80 days of the organic solvent had almost no effect on the membrane, with chemical groups on the surface of the PVA membrane.

### 3.4. Energy Consumption

#### Pervaporation Energy Consumption

Calculated for 1 ton as an example, the data for pervaporation are shown in Table 1.

**Figure 13:**
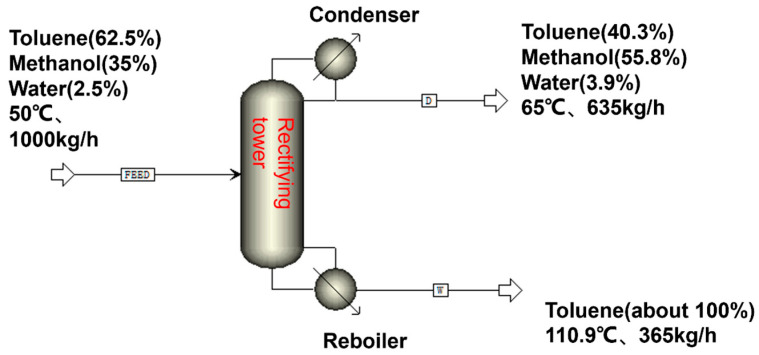
Process simulation.

Therefore, pervaporation requires an energy consumption of 43.53 kW·h to treat 1 ton of raw material, without taking into account the energy consumption of pumps and other power equipment. In order to demonstrate the energy-saving benefits of pervaporation, we conducted a simulated energy consumption comparison for distillation. The simulation is shown in Figure 13. Due to the azeotropy between methanol, toluene, and toluene water, a large amount of toluene must be evaporated if the water is to be evaporated.

We simulated the distillation process and found that the energy consumption for processing 1 ton of raw material is about 261.5 kW·h. The high energy consumption of distillation is mainly due to the ternary azeotropy of toluene/methanol/water that must be carried to evaporate most of the methanol and toluene during dehydration. This not only leads to an increase in energy consumption but also to a reduction in toluene yield. Pervaporation technology can overcome the above-mentioned disadvantages of distillation, so pervaporation technology is more advantageous. Compared to the present distillation process, the pervaporation process has significantly lower energy consumption (about one-sixth of the energy consumption of distillation).

## 4. Conclusions

In this paper, a method for the dehydration of organic solvents with reduced costs and energy consumption for a toluene/methanol/water mixture was proposed. In the pervaporation test, the results showed that the water/methanol separation factor, the methanol content in the feed out, and the water content in the permeate increased with temperature, flow rate, and vacuum degree, while the water and toluene content in the feed out and the methanol content in the permeate decreased with temperature, flow rate, and vacuum degree. At a temperature of 50 °C, a flow rate of 50 L/h, and a permeate side vacuum of 60 kPa, the water and methanol content in the permeate was about 63.2 wt.% and 36.8 wt.%, respectively, the water/methanol separation factor was 24.04, the permeate flux was 510.7 g/m^2^·h, the water content in the feed out was reduced from 2.5 wt.% to less than 0.66 wt.%, and the dehydration of toluene methanol could be realized. Without taking into account the energy consumption of pumps and other power equipment, pervaporation requires an energy consumption of 43.53 kW·h to treat 1 ton of raw material, while the energy consumption of distillation to treat 1 ton of raw material is about 261.5 kW·h. Compared to the existing distillation process, the pervaporation process uses significantly less energy (about one-sixth of the energy consumption of distillation). There is almost no effect on the surface morphology and chemical composition of the membrane before and after use. The method provides an effective reference for the dehydration of organic solvents from ternary mixtures containing toluene/methanol/water.

## Figures and Tables

**Table 1:** The parameters of the organic solvent.

	Water	Methanol	Toluene
Feed/kg	25	350	625
Feed out/kg	6.6	246.2	
Permeate/kg	18.4	103.8	
Δ*H*_ev_/J/g	2260	1109	
*Q*_pevap_/kW·h	18.4 × 2260 + 103.8 × 1109 = 156,698 kJ = 43.53 kW·h (1 kW·h = 3600 kJ)		

## Data Availability

The original contributions presented in the study are included in the article, further inquiries can be directed to the corresponding authors.
